# Contemporary Analysis of Reexcision and Conversion to Mastectomy Rates and Associated Healthcare Costs for Women Undergoing Breast-Conserving Surgery

**DOI:** 10.1245/s10434-024-14902-z

**Published:** 2024-02-06

**Authors:** Youngran Kim, Cecilia Ganduglia-Cazaban, Nina Tamirisa, Anthony Lucci, Trudy Millard Krause

**Affiliations:** 1 Department of Management, Policy and Community Health, The University of Texas Health Science Center at Houston School of Public Health, Houston, TX USA; 2 Department of Breast Surgical Oncology, The University of Texas MD Anderson Cancer Center, Houston, TX USA

**Keywords:** Breast cancer surgery, Breast-conserving surgery, Reoperation, Re-excision, Conversion to mastectomy, Healthcare costs, Medicare, Ductal carcinoma in situ

## Abstract

**Purpose:**

This study was designed to provide a comprehensive and up-to-date understanding of population-level reoperation rates and incremental healthcare costs associated with reoperation for patients who underwent breast-conserving surgery (BCS).

**Methods:**

This is a retrospective cohort study using Merative™ MarketScan^®^ commercial insurance data and Medicare 5% fee-for-service claims data. The study included females aged 18–64 years in the commercial cohort and females aged 18 years and older in the Medicare cohort, who underwent initial BCS for breast cancer in 2017–2019. Reoperation rates within a year of the initial BCS and overall 1-year healthcare costs stratified by reoperation status were measured.

**Results:**

The commercial cohort included 17,129 women with a median age of 55 (interquartile range [IQR] 49–59) years, and the Medicare cohort included 6977 women with a median age of 73 (IQR 69–78) years. Overall reoperation rates were 21.1% (95% confidence interval [CI] 20.5–21.8%) for the commercial cohort and 14.9% (95% CI 14.1–15.7%) for the Medicare cohort. In both cohorts, reoperation rates decreased as age increased, and conversion to mastectomy was more prevalent among younger women in the commercial cohort. The mean healthcare costs during 1 year of follow-up from the initial BCS were $95,165 for the commercial cohort and $36,313 for the Medicare cohort. Reoperations were associated with 24% higher costs in both the commercial and Medicare cohorts, which translated into $21,607 and $8559 incremental costs, respectively.

**Conclusions:**

The rates of reoperation after BCS have remained high and have contributed to increased healthcare costs. Continuing efforts to reduce reoperation need more attention.

**Supplementary Information:**

The online version contains supplementary material available at 10.1245/s10434-024-14902-z.

Breast cancer is the second most common cancer in women following skin cancer and accounts for approximately 30% of all new female cancers each year in the United States.^1^ Breast-conserving surgery (BCS) aims to remove the malignancy with preservation of the breast, and between 60–70% of those diagnosed with breast cancer undergo BCS.^2,3^ In BCS, the surgeon removes the cancerous tissue along with a surrounding margin of healthy tissue while preserving as much of the breast as possible. However, if cancer cells are present at the margins of the removed tissue, additional operations may be necessary. These additional operations may result in increased adverse outcomes, such as patient anxiety, surgical complications, treatment delays, and increasing healthcare costs.^4–7^

In 2014, the Society of Surgical Oncology-American Society for Radiation Oncology (SSO-ASTRO) Consensus Guideline on Margins for Breast-Conserving Surgery established a no-ink-on-tumor definition of a negative margin.^8^ The 2016 consensus guidelines further specified 2 mm as a negative margin for ductal carcinoma in situ (DCIS).^9,10^ Several studies demonstrated that reoperation rates substantially decreased as a result of the adoption of these clinical guidelines.^11–13^ However, despite the established guidelines,^8,9^ a significant number of patients continue to undergo reoperation with wide variations across different surgeons and institutions.^11,12,14–20^

In this study, we provide a more comprehensive and current understanding of reoperation rates and incremental healthcare costs associated with reoperation for patients who underwent BCS at the population level. Previous studies have primarily focused on the impact of guideline implementation on reoperation rates using institutional-level data and often excluded DICS cases, which accounts for approximately 20% of newly diagnosed cases.^10,21,22^ Moreover, younger women with DCIS are increasingly undergoing BCS procedures.^23,24^ However, achieving clear margins for DCIS can be challenging because of its nonpalpable nature and difficulty in preoperative disease extent delineation through imaging.^9,10,21^ Furthermore, the increasing use of neoadjuvant chemotherapy (NAC) before surgery and improved oncoplastic surgery (OPS) have impacted patient eligibility for BCS, potentially altering the characteristics of patients receiving BCS and their likelihood of receiving reoperation.^25–27^ Also, cost data are scarce and often are limited to surgical costs alone, without providing a comprehensive perspective on the overall cost of care for women undergoing BCS or relied on hospital charges, which can be different from the amount paid.^28–30^ Additionally, because of dated publications on the cost of reoperation, the impact of growing healthcare costs and the influence of cost containment strategies, such as bundling, have not been adequately accounted for in BCS cost studies.^7,31^

## Methods

### Study Design and Study Participants

We conducted a retrospective analysis on two sets of data: the 2016–2020 Merative™ MarketScan^®^ commercial insurance data, referred to as the “Commercial cohort,” and the 2016–2020 Medicare claims data, referred to as the “Medicare cohort.” The MarketScan database consisted of claims data from approximately 350 unique carriers and more than 273 million unique patients, including employees, spouses, and dependents from all U.S. census regions. The Medicare data was a nationally random 5% sample of all beneficiaries enrolled in Medicare fee-for-service (FFS). This study was approved by the institutional review board, and waivers of informed consent were granted due to the use of deidentified data. The study adhered to the Strengthening the Reporting of Observational Studies in Epidemiology (STROBE) reporting guideline.^32^

We included females aged 18–64 years who underwent initial BCS for breast cancer between January 1, 2017, and December 31, 2019 in the commercial cohort (*n* = 28,962). For the Medicare cohort, we included females aged 18 years and older who underwent initial BCS for breast cancer between January 1, 2017, and October 31, 2019 (*n* = 8380). Initial BCS was identified using Current Procedural Terminology (CPT) codes and *International Classification of Diseases, Tenth Revision, Procedure Coding System* (ICD-10-PCS) codes, which require the documentation of attention to surgical margins.^7,19,28^ Breast cancer was determined using the *International Classification of Diseases, Tenth Revision, Clinical Modification* (ICD-10-CM) codes (eTable 1 in the Supplement) and included cases of nonmetastatic breast cancer with a diagnosis of invasive breast cancer (IBC) or DCIS at the time of their initial BCS. To ensure that patients did not have previous BCS or mastectomy before the initial BCS and to determine reoperations and healthcare costs during the follow-up period, we required continuous enrollments between 1 year before and after the initial BCS. Patients who had any claims indicating previous BCS or mastectomy during the look-back period or had missing geographic information were excluded (eTable 2 in the Supplement).

### Outcomes

The primary outcomes were reoperation within a year of the initial BCS and overall 1-year healthcare costs stratified by reoperation status. The reoperation was defined as the presence of a procedural claim for repeated BCS or conversion to mastectomy identified using CPT and ICD-10-PCS codes (eTable 1 in the Supplement). The costs were calculated from a payer perspective and included amounts reimbursed by insurance and paid by the patient. The costs included those for outpatient and inpatient services and were categorized into breast surgical procedures, radiology treatment, chemotherapy, outpatient medication, pathology/laboratory, imaging, and others. All costs were inflation-adjusted using the medical care component of the Consumer Price Index and reported in 2020 U.S. dollars. The secondary outcomes included complications within 90 days, which were determined as having any claim with a diagnosis of infection, abscess, or cellulitis of the breast, or other postprocedural complications using ICD-10-CM diagnosis codes (eTable 3 in the Supplement).^26^

### Patient and Treatment Characteristics

The characteristics of the patients included age, race/ethnicity (available only for Medicare patients), census region of residence, cancer diagnosis, and Charlson Comorbidity Index (CCI) (eTable 4 in the Supplement).^33,34^ Cancer diagnosis was determined as IBC, DCIS, or mixed type for those with both IBC and DCIS diagnosis codes. The receipt of NAC was determined by having any claims with CPT and Healthcare Common Procedure Coding System (HCPCS) codes indicating adjuvant chemotherapy within 90 days before the initial BCS (eTable 5 in the Supplement).^35^ The receipt of OPS was determined by having any claims with CPT/HCPCS codes indicating oncoplasty or breast reconstruction during the follow-up time period (eTable 6 in the Supplement).^26,27^ If the OPS occurred on the same date of the initial BCS, we defined it as immediate OPS.

### Statistical Analysis

Reoperation rates were defined as proportions of patients who had repeated BCS or conversion to mastectomy within 1 year after the initial BCS and compared across patient characteristics using crude risk ratios (RRs). To assess independent associations between patient characteristics/treatment variables and reoperation, we performed multivariable modified Poisson regressions, including age, cancer diagnosis, CCI, NAC, immediate OPS, year of initial BCS, regions, and race/ethnicity (only for Medicare), and reported adjusted RRs. Mean costs following BCS were compared by reoperation status, and relative ratios (RRs) of cost were estimated from the generalized linear model with a log-link function and gamma distribution adjusting for the same variables to determine the incremental effect of reoperation on medical care costs. Similarly, complication rates were reported, and crude and adjusted RRs were reported by using modified Poisson regressions. The analyses were conducted separately for the Commercial cohort and Medicare cohorts. All statistical analyses were performed by using StataMP 17 (StataCorp LLC, College Station, TX) and significances were tested at *p* < 0.05 for two-tailed tests.

## Results

### Patients Characteristics and Treatments

In the Commercial cohort, 3620 of 17,129 women had reoperation, and in the Medicare cohort, 1039 of 6977 women had reoperation (Table 1). The prevalence of DCIS was 20.3% in the Commercial cohort, and it was higher among women with reoperation (17.8% vs. 29.5%). Similarly, the prevalence of DCIS was higher among women with reoperation (13.7% vs. 24.7%) in the Medicare cohort. One in eight women (13.5%) received NAC in the Commercial cohort, whereas only 6.7% had NAC in the Medicare cohort despite having a higher prevalence of IBC (68.6% in Commercial vs. 77.1% in Medicare, p < 0.001). Approximately one in four women (27.2%) in the Commercial cohort and one in five women (18.6%) in the Medicare cohort had OPS, and most of the OPS were performed on the same day of the initial BCS.

**Table 1:** Characteristics by reoperation status: Commercial and Medicare cohorts

Characteristics, No. (%)	Commercial cohort							Medicare cohort						
					Among any reoperation							Among any reoperation		
	All	No reoperation	Any reoperation	*p*	Repeated BCS	Convert to mastectomy	*p*	All	No reoperation	Any reoperation	*p*	Repeated BCS	Convert to mastectomy	*p*
No. of Patients	17,129	13,509	3620		2563	1057		6977	5938	1039		771	268	
*Age, years*														
Mean (SD)	53.7 (7.0)	53.9 (6.9)	53.0 (7.3)	<0.001	53.7 (7.0)	51.2 (7.7)	<0.001	73.5 (7.3)	73.6 (7.3)	72.4 (7.4)	<0.001	72.5 (6.9)	72.0 (8.4)	0.34
Median [IQR]	55 [49–59]	55 [49–60]	54 [48–59]	<0.001	55 [49–60]	52 [46–58]	<0.001	73 [69–78]	73 [69–78]	72 [68–77]	<0.001	72 [68–76]	71.5 [68–77]	0.67
*Diagnosis*														
IBC	11,747 (68.6)	9633 (71.3)	2114 (58.4)	<0.001	1526 (59.5)	588 (55.6)	0.038	5382 (77.1)	4697 (79.1)	685 (65.9)	<0.001	506 (65.6)	179 (66.8)	0.88
DCIS	3474 (20.3)	2405 (17.8)	1069 (29.5)		747 (29.1)	322 (30.5)		1071 (15.4)	814 (13.7)	257 (24.7)		191 (24.8)	66 (24.6)	
Mixed	1908 (11.1)	1471 (10.9)	437 (12.1)		290 (11.3)	147 (13.9)		524 (7.5)	427 (7.2)	97 (9.3)		74 (9.6)	23 (8.6)	
*Index Year*														
2017	5639 (32.9)	4430 (32.8)	1209 (33.4)	0.079	855 (33.4)	354 (33.5)	0.92	2312 (33.1)	1950 (32.8)	362 (34.8)	0.42	276 (35.8)	86 (32.1)	0.54
2018	5638 (32.9)	4408 (32.6)	1230 (34.0)		867 (33.8)	363 (34.3)		2472 (35.4)	2109 (35.5)	363 (34.9)		266 (34.5)	97 (36.2)	
2019	5852 (34.2)	4671 (34.6)	1181 (32.6)		841 (32.8)	340 (32.2)		2193 (31.4)	1879 (31.6)	314 (30.2)		229 (29.7)	85 (31.7)	
*Region*														
Northeast	3819 (22.3)	3014 (22.3)	805 (22.2)	0.37	586 (22.9)	219 (20.7)	0.002	1387 (19.9)	1205 (20.3)	182 (17.5)	0.044	136 (17.6)	46 (17.2)	0.20
Midwest	3528 (20.6)	2818 (20.9)	710 (19.6)		488 (19.0)	222 (21.0)		1526 (21.9)	1269 (21.4)	257 (24.7)		203 (26.3)	54 (20.1)	
South	7496 (43.8)	5877 (43.5)	1619 (44.7)		1175 (45.8)	444 (42.0)		2693 (38.6)	2294 (38.6)	399 (38.4)		288 (37.4)	111 (41.4)	
West	2286 (13.3)	1800 (13.3)	486 (13.4)		314 (12.3)	172 (16.3)		1371 (19.7)	1170 (19.7)	201 (19.3)		144 (18.7)	57 (21.3)	
*CCI*														
0–1	13,596 (79.4)	10,624 (78.6)	2972 (82.1)	<0.001	2101 (82.0)	871 (82.4)	0.64	3914 (56.1)	3305 (55.7)	609 (58.6)	0.19	463 (60.1)	146 (54.5)	0.13
2–5	1940 (11.3)	1567 (11.6)	373 (10.3)		271 (10.6)	102 (9.6)		2305 (33.0)	1978 (33.3)	327 (31.5)		239 (31.0)	88 (32.8)	
>5	1593 (9.3)	1318 (9.8)	275 (7.6)		191 (7.5)	84 (7.9)		758 (10.9)	655 (11.0)	103 (9.9)		69 (8.9)	34 (12.7)	
NAC	2314 (13.5)	1977 (14.6)	337 (9.3)	<0.001	229 (8.9)	108 (10.2)	0.23	468 (6.7)	424 (7.1)	44 (4.2)	<0.001	26 (3.4)	18 (6.7)	0.019
OPS	4662 (27.2)	3301 (24.4)	1361 (37.6)	<0.001	598 (23.3)	763 (72.2)	<0.001	1297 (18.6)	1032 (17.4)	265 (25.5)	<0.001	141 (18.3)	124 (46.3)	<0.001
Immediate OPS	3656 (21.3)	3006 (22.3)	650 (18.0)	<0.001	418 (16.3)	232 (21.9)	<0.001	1167 (16.7)	1003 (16.9)	164 (15.8)	0.38	122 (15.8)	42 (15.7)	0.95
*Complications*														
Any	3463 (20.2)	2391 (17.7)	1072 (29.6)	<0.001	736 (28.7)	336 (31.8)	0.066	1313 (18.8)	972 (16.4)	341 (32.8)	<0.001	231 (30.0)	110 (41.0)	<0.001
Fat necrosis	879 (5.1)	452 (3.3)	427 (11.8)	<0.001	328 (12.8)	99 (9.4)	0.004	360 (5.2)	212 (3.6)	148 (14.2)	<0.001	115 (14.9)	33 (12.3)	0.29
Infection	769 (4.5)	504 (3.7)	265 (7.3)	<0.001	190 (7.4)	75 (7.1)	0.74	371 (5.3)	270 (4.5)	101 (9.7)	<0.001	72 (9.3)	29 (10.8)	0.48
Hemorrhage	629 (3.7)	426 (3.2)	203 (5.6)	<0.001	131 (5.1)	72 (6.8)	0.043	338 (4.8)	245 (4.1)	93 (9.0)	<0.001	64 (8.3)	29 (10.8)	0.21
Deformity	969 (5.7)	727 (5.4)	242 (6.7)	0.003	142 (5.5)	100 (9.5)	<0.001	222 (3.2)	172 (2.9)	50 (4.8)	0.001	26 (3.4)	24 (9.0)	<0.001
Breast pain	410 (2.4)	317 (2.3)	93 (2.6)	0.44	58 (2.3)	35 (3.3)	0.07	174 (2.5)	139 (2.3)	35 (3.4)	0.05	24 (3.1)	11 (4.1)	0.44
Other	767 (4.5)	568 (4.2)	199 (5.5)	<0.001	124 (4.8)	75 (7.1)	0.007	253 (3.6)	194 (3.3)	59 (5.7)	<0.001	37 (4.8)	22 (8.2)	0.038

*BCS* breast-conserving surgery; *SD* standard deviation; *IQR* interquartile range; *IBC* invasive breast cancer; *DCIS* ductal carcinoma in situ; *CCI* Charlson Comorbidity Index; *NAC* neoadjuvant chemotherapy; *OPS* oncoplastic surgery

*Differences in patient characteristics across reoperation status were assessed and *p* values were reported from chi-square tests for categorical variables and Wilcoxon rank-sum tests for continuous variables

### Reoperation Rates

**Fig. 1:**
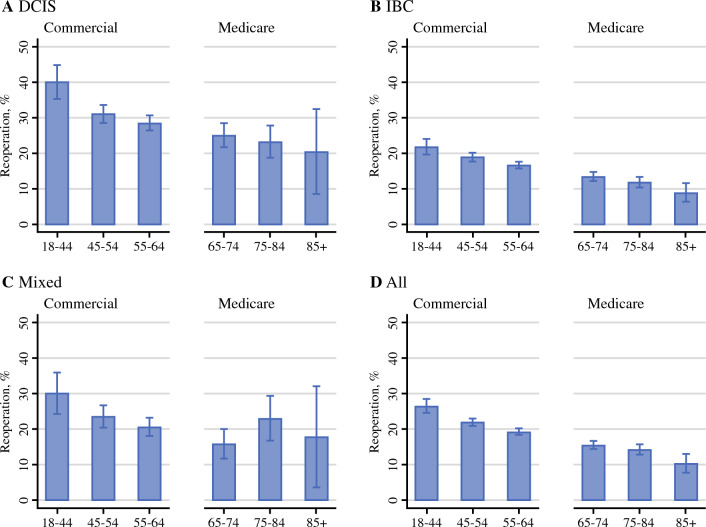
Reoperation rates by age group and cancer diagnosis. Proportions of patients who had repeated breast-conserving surgery (BCS) or conversion to mastectomy within 1 year after the initial BCS were graphed by age and cancer diagnosis: invasive breast cancer (IBC), ductal carcinoma in situ, or mixed. Rates were estimated separately for the Commercial and Medicare cohorts. Patients younger than aged 65 years in the Medicare cohort were not included. Error bars indicate 95% confidence intervals

Overall reoperation rates were 21.1% (95% confidence interval [CI] 20.5–21.8%) for the Commercial cohort and 14.9% (95% CI 14.1–15.7%) for the Medicare cohort (Table 2). In the Commercial cohort, reoperation rates decreased as the age increased: 26.5%, 22.0%, and 19.4% (*p* value for trend <0.001) for aged 18–44, 45–54, and 55–64 years, respectively (Table 2). While the rates of repeated BCS were similar across age groups, the conversion to mastectomy was more prevalent among younger women in the Commercial cohort. In the Medicare cohort, women aged 18–64 years had an 18.6% (95% CI 14.8–23.1%) reoperation rate, which was comparable to that of women in the same age group in the Commercial cohort. Additionally, a similar downward trend by age was observed, but it did not reach a significant level (Table 2). When we stratified the analysis by cancer diagnosis and age (Fig. 1), we observed similar downward trends by age across cancer diagnoses.

**Table 2:** Reoperation rates by subgroup

	No.	Any reoperation, %	*p*	Repeated BCS, %	*p*	Conversion to mastectomy, %	*p*
Commercial, total	3620/17,129	21.1 (20.5–21.8)		15.0 (14.4–15.5)		6.2 (5.8–6.5)	
*Age (years)*							
18–44	519/1955	26.5 (24.6–28.6)	<0.001	15.1 (13.6–16.8)	0.48	11.4 (10.1–12.9)	<0.001
45–54	1361/6184	22.0 (21.0–23.1)		15.2 (14.3–16.1)		6.8 (6.2–7.4)	
55–64	1740/8990	19.4 (18.6–20.2)		14.7 (14.0–15.5)		4.6 (4.2–5.1)	
*Diagnosis*							
IBC	2114/11,747	18.0 (17.3–18.7)	<0.001	13.0 (12.4–13.6)	<0.001	5.0 (4.6–5.4)	<0.001
DCIS	1069/3474	30.8 (29.3–32.3)		21.5 (20.2–22.9)		9.3 (8.3–10.3)	
Mixed	437/1908	22.9 (21.1–24.8)		15.2 (13.7–16.9)		7.7 (6.6–9.0)	
*Diagnosis x age*							
IBC, 18–44	287/1316	21.8 (19.7–24.1)	<0.001	12.8 (11.1–14.8)	0.92	9.0 (7.5–10.6)	<0.001
45–54	788/4170	18.9 (17.7–20.1)		13.1 (12.1–14.2)		5.8 (5.1–6.5)	
55–64	1039/6261	16.6 (15.7–17.5)		12.9 (12.1–13.8)		3.7 (3.2–4.2)	
DCIS, 18–44	160/400	40.0 (35.3–44.9)	<0.001	21.5 (17.7–25.8)	0.76	18.5 (15.0–22.6)	<0.001
45–54	406/1306	31.1 (28.6–33.7)		21.9 (19.7–24.2)		9.2 (7.7–10.9)	
55–64	503/1768	28.5 (26.4–30.6)		21.2 (19.4–23.2)		7.2 (6.1–8.5)	
Mixed, 18–44	72/239	30.1 (24.6–36.2)	0.002	17.2 (12.9–22.5)	0.42	13.0 (9.3–17.9)	<0.001
45–54	167/708	23.6 (20.6–26.9)		15.1 (12.7–17.9)		8.5 (6.6–10.8)	
55–64	198/961	20.6 (18.2–23.3)		14.8 (12.7–17.2)		5.8 (4.5–7.5)	
*NAC*							
No	3283/14,815	22.2 (21.5–22.8)	<0.001	15.8 (15.2–16.3)	<0.001	6.4 (6.0–6.8)	0.001
Yes	337/2314	14.6 (13.2–16.1)		9.9 (8.7–11.2)		4.7 (3.9–5.6)	
*Immediate OPS*							
No	2970/13,473	22.0 (21.4–22.8)	<0.001	15.9 (15.3–16.5)	<0.001	6.1 (5.7–6.5)	0.62
Yes	650/3656	17.8 (16.6–19.1)		11.4 (10.4–12.5)		6.3 (5.6–7.2)	
Medicare, total	1039/6977	14.9 (14.1–15.7)		11.1 (10.3–11.8)		3.8 (3.4–4.3)	
*Age (years)*							
18–64	63/339	18.6 (14.8–23.1)	<0.001	12.7 (9.5–16.7)	0.002	5.9 (3.8–9.0)	0.14
65–74	595/3832	15.5 (14.4–16.7)		11.7 (10.8–12.8)		3.8 (3.2–4.4)	
75–84	327/2286	14.3 (12.9–15.8)		10.5 (9.3–11.8)		3.9 (3.1–4.7)	
85+	54/520	10.4 (8.0–13.3)		7.5 (5.5–10.1)		2.9 (1.8–4.7)	
*Diagnosis*							
IBC	685/5382	12.7 (11.9–13.6)	<0.001	9.4 (8.7–10.2)	<0.001	3.3 (2.9–3.8)	<0.001
DCIS	257/1071	24.0 (21.5–26.6)		17.8 (15.7–20.2)		6.2 (4.9–7.8)	
Mixed	97/524	18.5 (15.4–22.1)		14.1 (11.4–17.4)		4.4 (2.9–6.5)	
*Diagnosis x age*							
IBC, 18–64	45/251	17.9 (13.7–23.2)	<0.001	12.0 (8.5–16.6)	0.001	6.0 (3.6–9.7)	0.14
65–74	390/2902	13.4 (12.2–14.7)		10.2 (9.1–11.3)		3.3 (2.7–4.0)	
75–84	210/1781	11.8 (10.4–13.4)		8.7 (7.4–10.0)		3.1 (2.4–4.1)	
85+	40/448	8.9 (6.6–11.9)		6.0 (4.2–8.7)		2.9 (1.7–4.9)	
DCIS, 18–64	13/63	20.6 (12.4–32.4)	0.69	15.9 (8.8–27.1)	0.68	4.8 (1.5–13.8)	0.96
65–74	157/628	25.0 (21.8–28.5)		18.6 (15.8–21.9)		6.4 (4.7–8.6)	
75–84	78/336	23.2 (19.0–28.0)		17.0 (13.3–21.4)		6.3 (4.1–9.4)	
85+	9/44	20.5 (11.0–34.9)		15.9 (7.8–29.8)		4.6 (1.1–16.4)	
Mixed, 18–64	5/25	20.0 (8.6–40.0)	0.24	12.0 (3.9–31.3)	0.20	8.0 (2.0–27.0)	0.94
65–74	48/302	15.9 (12.2–20.5)		12.6 (9.3–16.8)		3.3 (1.8–6.0)	
75–84	39/169	23.1 (17.3–30.0)		16.6 (11.7–23.0)		6.5 (3.6–11.4)	
85+	5/28	17.9 (7.6–36.4)		17.9 (7.6–36.4)			
*NAC*							
No	995/6509	15.3 (14.4–16.2)	0.001	11.5 (10.7–12.2)	<0.001	3.8 (3.4–4.3)	0.99
Yes	44/468	9.4 (7.1–12.4)		5.6 (3.8–8.0)		3.9 (2.4–6.0)	
*Immediate OPS*							
No	875/5810	15.1 (14.2–16.0)	0.38	11.2 (10.4–12.0)	0.48	3.9 (3.4–4.4)	0.64
Yes	164/1167	14.1 (12.2–16.2)		10.5 (8.8–12.3)		3.6 (2.7–4.8)	

*BCS* breast-conserving surgery; *IBC* invasive breast cancer; *DCIS* ductal carcinoma in situ; *NAC* neoadjuvant chemotherapy; *OPS* oncoplastic surgery

*p values were reported from the chi-square tests except for age-specific and cancer diagnosis and age-specific reoperation rates, which were tested for a linear trend by using Cochran–Armitage tests

Compared with women with IBC, women with DCIS had higher reoperation rates: 18.0% vs. 30.8%, *p* < 0.001 in the commercial cohort and 12.7% vs. 24.0%, *p* < 0.001 in the Medicare cohort. Notably, the highest rate of reoperation, 40.0% (95% CI 35.3–44.9%), and particularly the highest rate of conversion to mastectomy were found among women aged 18–44 years with DCIS (Table 2). In multivariable analyses, compared with IBC, DCIS was associated with a 62% increased risk of reoperation {adjusted relative risk [aRR] 1.62 (1.52–1.73)} in the Commercial cohort and an 80% increased risk [aRR 1.80 (1.58–2.05)] in the Medicare cohort (eTables 7 and 8 in the Supplement). NAC was associated with a 29% decreased risk of reoperation (aRR 0.71 [95% CI 0.64–0.80]) in the Commercial cohort and a 35% decreased risk (aRR 0.65 [95% CI 0.48–0.88]) in the Medicare cohort. Immediate OPS was associated with lower reoperation rates in the Commercial cohort (aRR 0.83 [95% CI 0.77–0.89]), but not in the Medicare cohort.

### Complication Rates by Reoperation Status

**Fig. 2:**
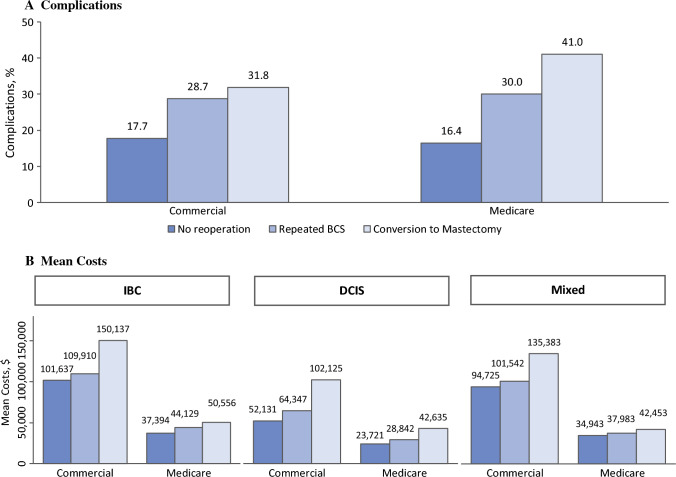
Rates of complications and 1-year healthcare costs by reoperation status. Complications included infection, abscess, or cellulitis of the breast, or other postprocedural complications within 90 days. Overall 1-year healthcare costs were calculated from a payer perspective and included amounts reimbursed by insurance and paid by the patient for breast surgical procedures, radiology treatment, chemotherapy, outpatient medication, pathology/laboratory, imaging, and others. All costs were inflation-adjusted using the medical care component of the Consumer Price Index and reported in 2020 U.S. dollars

Approximately one in five women who underwent BCS experienced complications within 90 days (eTables 9 and 10 in the Supplement), and complication rates were much higher among women with reoperation in both cohorts (Fig. 2A). Compared with no reoperation, reoperation was associated with a 54% increased risk of complications (aRR 1.54, 95% CI 1.44–1.64) in the Commercial cohort and an 89% increased risk (aRR 1.89, 95% CI 1.70–2.10) in the Medicare cohort adjusting for covariates. Repeated BCS was associated with a 66% increased risk of complications (aRR 1.66, 95% CI 1.55–1.78) in the Commercial cohort and an 82% increased risk (aRR 1.82, 95% CI 1.61–2.06) in the Medicare cohort (eTables 9 and 10 in the Supplement). Conversion to mastectomy was associated with a 30% increased risk (aRR 1.30, 95% CI 1.18–1.44) in the Commercial cohort, whereas it was associated with more than a twofold increased risk (aRR 2.07, 95% CI 1.77–2.44) in the Medicare cohort (eTables 9 and 10 in the Supplement).

### Healthcare Costs by Reoperation Status

The mean healthcare costs during 1 year of follow-up from the initial BCS were $95,165 for the Commercial cohort and $36,313 for the Medicare cohort. These costs varied substantially based on cancer diagnosis and reoperation status (Fig. 2). Specifically, costs were significantly lower for women with DCIS compared with those with IBC or mixed diagnoses. In the Commercial cohort, costs for women with DCIS were almost half of those with IBC (aRR 0.57, 95% CI 0.55–0.59). In the Commercial cohort, the costs were 14% higher (aRR 1.14, 95% CI 1.10–1.18) with an incremental cost of $12,638 (SE $1954) for women with repeated BCS and 51% higher (aRR 1.51, 95% CI 1.42–1.60) with an incremental cost of $45,989 (SE $3979) for women with conversion to mastectomy, compared with women without reoperation, adjusting for covariates (Table 3). In the Medicare cohort, the costs were 19% higher (aRR 1.19, 95% CI 1.12–1.27) with an incremental cost of $6742 (SE $1367) for women with repeated BCS and 40% higher (aRR 1.40, 95% CI 1.26–1.56) with an incremental cost of $12,638 (SE $1954) for women with conversion to mastectomy (Table 3). Combining repeated BCS and conversion to mastectomy as reoperation, the costs for women with reoperation were associated with 24% higher healthcare costs in both the Commercial (aRR 1.24, 95% CI 1.20–1.28) and Medicare (aRR 1.24, 95% CI 1.17–1.32) cohorts, resulting into $21,607 (SE $1853) and $8559 (SE $1251) incremental costs associated with reoperation, respectively.

**Table 3:** Mean 1-year healthcare costs and relative ratios by subgroup

	Commercial Cohort					Medicare cohort				
	Mean Cost^a^, $	Crude RR	*p*	Adjusted RR^b^	*p*	Mean cost^a^, $	Crude RR	*p*	Adjusted RR^b^	*p*
All	95,165					36,313				
*Reoperation*										
No	92,071	1.00		1.00		35,343	1.00		1.00	
Repeated BCS	95,684	1.04 (1.00–1.08)	0.06	1.14 (1.10–1.18)	<0.001	39,752	1.12 (1.05–1.20)	0.001	1.19 (1.12–1.27)	<0.001
Conversion to mastectomy	133,459	1.45 (1.37–1.54)	<0.001	1.51 (1.42–1.60)	<0.001	47,910	1.36 (1.21–1.52)	<0.001	1.40 (1.26–1.56)	<0.001
*Age*^*c*^										
18–44	117,323	1.00		1.00						
45–54	96,936	0.83 (0.79–0.87)	<0.001	0.89 (0.85–0.93)	<0.001					
55–64	89,129	0.76 (0.73–0.80)	<0.001	0.83 (0.80–0.87)	<0.001					
18–64						48,233	1.00		1.00	
65–74						38,549	0.80 (0.72–0.88)	<0.001	0.87 (0.79–0.95)	0.003
75–84						33,313	0.69 (0.62–0.77)	<0.001	0.73 (0.66–0.81)	<0.001
85+						25,256	0.52 (0.46–0.59)	<0.001	0.56 (0.49–0.63)	<0.001
*Diagnosis*										
IBC	105,139	1.00		1.00		38,465	1.00		1.00	
DCIS	59,392	0.56 (0.55–0.58)	<0.001	0.57 (0.55–0.59)	<0.001	25,800	0.67 (0.63–0.71)	<0.001	0.67 (0.63–0.71)	<0.001
Mixed	98,894	0.94 (0.90–0.98)	0.006	0.94 (0.90–0.98)	0.007	35,702	0.93 (0.86–1.01)	0.068	0.93 (0.86–1.00)	0.059
*CCI*										
0–1	89,250	1.00		1.00		32,463	1.00		1.00	<0.001
2–5	96,968	1.09 (1.04–1.14)	<0.001	1.10 (1.05–1.15)	<0.001	37,454	1.15 (1.10–1.21)	<0.001	1.17 (1.12–1.22)	<0.001
>5	143,453	1.61 (1.53–1.69)	<0.001	1.31 (1.24–1.38)	<0.001	52,726	1.62 (1.52–1.74)	0.00	1.50 (1.40–1.61)	<0.001
*NAC*										
No	88,596	1.00		1.00		34,800	1.00		1.00	
Yes	137,228	1.55 (1.49–1.61)	<0.001	1.30 (1.24–1.36)	<0.001	57,352	1.65 (1.51–1.79)	<0.001	1.39 (1.28–1.51)	<0.001
*OPS*										
No	89,572	1.00		1.00		35,523	1.00		1.00	
Yes	110,122	1.23 (1.19–1.27)	<0.001	1.14 (1.10–1.17)	<0.001	39,772	1.12 (1.06–1.18)	<0.001	1.06 (1.00–1.12)	0.035
*Region*										
Northeast	110,134	1.00		1.00		36,116	1.00		1.00	
Midwest	91,084	0.83 (0.79–0.86)	<0.001	0.81 (0.77–0.84)	<0.001	35,923	0.99 (0.93–1.06)	0.87	0.97 (0.91–1.03)	0.33
South	87,759	0.80 (0.77–0.83)	<0.001	0.77 (0.74–0.79)	<0.001	36,763	1.02 (0.96–1.08)	0.56	0.96 (0.91–1.02)	0.18
West	100,746	0.91 (0.87–0.96)	<0.001	0.86 (0.82–0.90)	<0.001	36,063	1.00 (0.93–1.07)	0.97	0.98 (0.92–1.05)	0.61
*Race/ethnicity*^*d*^										
White						35,510	1.00		1.00	
Black						43,885	1.24 (1.14–1.34)	<0.001	1.16 (1.07–1.26)	<0.001
Hispanic						40,456	1.14 (1.01–1.29)	0.04	1.05 (0.94–1.19)	0.37
Other						35,653	1.00 (0.90–1.12)	0.94	0.96 (0.87–1.07)	0.49

*RR* relative ratio of costs; *IBC* invasive breast cancer; *DCIS* ductal carcinoma in situ; *CCI* Charlson Comorbidity Index; *NAC* neoadjuvant chemotherapy; *OPS* oncoplastic surgery

^a^Observed mean costs were inflation adjusted using the medical care component of Consumer Price Index and reported in 2020 U.S. dollars

^b^Adjusted cost ratios were reported from generalized linear models with the log-link function and gamma distribution including variables of reoperation status, age group, cancer diagnosis, CCI, NAC, OPS, Region, and year (race as well for the Medicare cohort only)

^c^Be noted that available age groups are different for the Commercial and Medicare cohorts

^d^Race/ethnicity was available only for the Medicare cohort

Older age and DCIS were independently associated with lower costs, whereas higher CCI, NAC, and OPS were independently associated with increased costs (Table 3). We also observed geographic variations with the lowest costs in the south in the Commercial cohort but did not observe such variations in the Medicare cohort. Black women had higher costs compared with white women, adjusting for covariates (aRR 1.16 [95% CI 1.07–1.26]) in the Medicare cohort. When we plotted costs by cancer diagnosis subgroup and by service category, incremental costs associated with reoperation appeared to be mostly driven by breast surgery costs. Furthermore, major differences in the costs by cancer diagnosis between IBC and DCIS were explained by differences in chemotherapy treatments (eFig. 1 in the Supplement).

## Discussion

The reoperation rates we observed were 21.1% for the Commercial cohort and 14.9% for the Medicare cohort, which were consistent with those reported in previous population-based studies, ranging from 14% to 22%.^11,12,14–16^ Although several studies reported a significant decrease in reoperation rates immediately after the publication of the guidelines, there was no evidence of further reduction observed in subsequent years. Because our study covered more recent years in the post-guideline period, it is expected that if the downward trend continued, we would have observed even lower reoperation rates. Several studies have found notable variations in the practices and reoperation rates among different surgeons and institutions that may indicate slower adoption of the guidelines and potentially avoidable reoperations.^11,12,17–20,36,37^ Promoting practice of standardized guidelines and improved intraoperative assessment of the surgical margins assisted by in real-time imaging techniques may have the potential to reduce avoidable reoperations.^38–40^

We observed downward trends in reoperation rates by age.^19^ This downward trend was primarily driven by a reduced conversion to mastectomy rate in the commercial cohort, which consisted of women younger than age 65 years. We found that the rate of conversion to mastectomy among women with DCIS was 9.3% in the Commercial cohort and younger women aged 18–44 years with DCIS had a reoperation rate of approximately 40%, with a high rate of conversion to mastectomy (18.5%). These findings are consistent with previous studies that have shown no decline in reexcision rates after BCS for patients with DCIS, along with increasing trends in choosing mastectomy as a conversion option.^41–43^ However, in the Medicare cohort where the majority of women were aged 65 years and older, the downward trend by age was driven by a reduction in repeated BCS. These findings suggest that older patients, particularly those with low-risk DCIS, are less likely to undergo reexcision compared with their younger counterparts, perhaps due to a lack of recurrence or survival benefit. It is important to note that more BCS procedures are being performed on DCIS patients, especially younger patients,^23,24^ and reoperation is more problematic as it is challenging to achieve clear margins.^9,10,21^

The use of NAC was 13.5% in the Commercial cohort and 6.7% in the Medicare cohort reflecting an increasing trend in NAC utilization, consistent with prior studies.^44–46^ We found that NAC was associated with a 29% decreased risk of reoperations in the Commercial cohort and a 35% decreased risk in the Medicare cohort. Lower rates of reoperation are expected in this subset of patients as tumor response to neoadjuvant therapy allows for patients to become better candidates for BCS with a higher chance of achieving negative margins.^47^ However, intraoperative palpation of the tumor bed or radiologic distortion on specimen radiography may not be a clear indication of residual disease in neoadjuvant chemotherapy patients, and optimal intraoperative assessment of margins becomes crucial, as it may be less obvious to identify clear margins post-NAC.

We confirmed that reoperation is associated with higher rates of complications and increased healthcare costs.^6,7^ In both Commercial and Medicare cohorts, we observed 54% and 89% increased risks of complications, respectively, among patients who underwent reoperation. Furthermore, reoperations were found to be associated with incremental costs of $21,607 in the Commercial cohort and $8559 in the Medicare cohorts. Among women who underwent conversion to mastectomy in the Commercial cohort, the incremental costs were particularly high, reaching $45,989. Interestingly, the mean cost for women who underwent BCS with or without reoperation in the Medicare cohort was less than half that of women in the Commercial cohort. Several factors may contribute to this difference, including the lower rate of reoperation, relatively lower reimbursement rates, and the implementation of value-based or global payment strategies. Additionally, because the Medicare cohort consists mostly of older patients, the downward trends by age likely contributed to the overall lower rate of reoperation. Notably, the rate of conversion to mastectomy, which is a more costly procedure, was low in the Medicare cohort.

### Limitations

This study has several limitations. First, we relied on ICD-10-CM codes to identify and define breast cancer, which limited the availability of detailed information regarding a patient’s cancer diagnosis, including stage at diagnosis. Consequently, we were unable to restrict our cohort to patients with ductal DCIS and stage I to II breast cancer. Second, our study did not incorporate clinical patient factors and tumor characteristics, which could have provided additional insights. Third, as we employed the same CPT codes for both the initial BCS and subsequent BCS procedures, it is possible that some of the reexcisions included in the study may have actually been the initial excision procedures for a different primary breast tumor. Lastly, race/ethnicity information was not available in the dataset for the Commercial cohort, which could have been a confounding factor as well.

## Conclusions

Overall reoperation rates after the initial BCS were 21.1% and 14.9% for the Commercial and Medicare cohorts, respectively, during 2017–2020. We found that younger age and DCIS were associated with a higher reoperation rate, while NAC and immediate OPS were associated with a lower reoperation rate. Reoperations were associated with 54% and 89% increased risk of complications in the Commercial and Medicare cohorts, respectively, and were associated with 24% increased costs in both cohorts.

### Supplementary Information

Below is the link to the electronic supplementary material.

Supplementary file1 (DOCX 146 KB)

## Data Availability

Data available: No. Additional Information: Explanation for why data not available: Our data were accessed as part of a data use agreement with the data owner, which prohibits access to the data for those not on the original agreement.
