# Correction: Contemporary Analysis of Reexcision and Conversion to Mastectomy Rates and Associated Healthcare Costs for Women Undergoing Breast-Conserving Surgery

**DOI:** 10.1245/s10434-024-15212-0

**Published:** 2024-03-23

**Authors:** Youngran Kim, Cecilia Ganduglia-Cazaban, Nina Tamirisa, Anthony Lucci, Trudy Millard Krause

**Affiliations:** 1 Department of Management, Policy and Community Health, The University of Texas Health Science Center at Houston School of Public Health, Houston, TX USA; 2 Department of Breast Surgical Oncology, The University of Texas MD Anderson Cancer Center, Houston, TX USA

**Correction: Annals of Surgical Oncology** 10.1245/s10434-024-14902-z

The article “Contemporary Analysis of Reexcision and Conversion to Mastectomy Rates and Associated Healthcare Costs for Women Undergoing Breast‑Conserving Surgery”, written by Kim et al. was originally published electronically on the publisher’s internet portal on February 6, 2024, without open access. With the authors‘ decision to opt for Open Choice the copyright of the article changed on March 14, 2024, to © The Author(s) 2024, and the article is forthwith distributed under a Creative Commons Attribution 4.0 International License, which permits use, sharing, adaptation, distribution and reproduction in any medium or format, as long as you give appropriate credit to the original author(s) and the source, provide a link to the Creative Commons licence, and indicate if changes were made. The images or other third party material in this article are included in the article’s Creative Commons licence, unless indicated otherwise in a credit line to the material. If material is not included in the article’s Creative Commons licence and your intended use is not permitted by statutory regulation or exceeds the permitted use, you will need to obtain permission directly from the copyright holder. To view a copy of this licence, visit http://creativecommons.org/licenses/by/4.0.

