# Supplementary material for: Contemporary Analysis of Reexcision and Conversion to Mastectomy Rates and Associated Healthcare Costs for Women Undergoing Breast-Conserving Surgery
Source: Ann Surg Oncol. 2024 Feb 6;31(6):3649–60. doi: 10.1245/s10434-024-14902-z (PMC11076367; doi:10.1245/s10434-024-14902-z)
Supplement: Supplementary file 1 — Supplementary file1 (DOCX 146 KB) [file 10434_2024_14902_MOESM1_ESM.docx]

**Supplemental Online Content**

**eTable 1.** Codes to identify initial breast-conserving surgery (BCS), reoperations, and breast cancer

**eTable 2.** Construction of Study Cohorts

**eTable 3.** Codes to identify complications

**eTable 4.** Codes to identify Charlson Comorbidities excluding breast cancer

**eTable 5.** CPT/HCPCS and revenue codes to identify the category of services

**eTable 6.** CPT/HCPCS Codes to identify oncoplasty and breast reconstruction

**eTable 7.** Reoperation rates by subgroup and risk ratios: Commercial cohort

**eTable 8.** Reoperation rates by subgroup and risk ratios: Medicare cohort

**eTable 9.** Complication rates by subgroup and risk ratios: Commercial cohort

**eTable 10.** Complication rates by subgroup and risk ratios: Medicare cohort

**eFigure 1.** Costs by Reoperation Status and Service type

**eTable 1. Codes to identify initial breast-conserving surgery (BCS), reoperations, and breast cancer**

| **Category** | **Code Type** | **Code** | **Description** |
| --- | --- | --- | --- |
| **Initial BCS** | CPT | 19301 | Partial mastectomy |
|  |  | 19302 | Partial mastectomy with axillary dissection |
|  | ICD-10-PCS | 0HBT0ZZ | Excision of right breast, open approach |
|  |  | 0HBU0ZZ | Excision of left breast, open approach |
|  |  | 0HBV0ZZ | Excision of bilateral breast, open approach |
| **Reoperation**: Repeated BCS | CPT | 19120 | Excision of breast tumor |
|  |  | 19125 | Excision of marked breast tumor |
|  |  | 19126 | Excision of marked breast tumor- each additional lesion |
|  |  | 19301 | Partial mastectomy |
|  |  | 19302 | Partial mastectomy with axillary dissection |
|  | ICD-10-PCS | 0HBT0ZX | Excision of right breast, open approach, diagnostic |
|  |  | 0HBU0ZX | Excision of left breast, open approach, diagnostic |
|  |  | 0HBV0ZX | Excision of bilateral breast, open approach, diagnostic |
|  |  | 0HBT0ZZ | Excision of right breast, open approach |
|  |  | 0HBU0ZZ | Excision of left breast, open approach |
|  |  | 0HBV0ZZ | Excision of bilateral breast, open approach |
| **Reoperation**: Conversion to mastectomy | CPT | 19303 | Mastectomy, simple, complete |
|  |  | 19304 | Mastectomy, subcutaneous |
|  |  | 19305 | Mastectomy, radical |
|  |  | 19306 | Mastectomy, radical |
|  |  | 19307 | Mastectomy, modified radical, |
|  | ICD-10-PCS | 0HTT0ZZ | Resection of right breast, open approach |
|  |  | 0HTU0ZZ | Resection of right breast, open approach |
|  |  | 0HTV0ZZ | Resection of Bilateral Breast by open approach |
| **Breast Cancer** | ICD-10-CM | D05.1x | Intraductal carcinoma in situ of breast (DCIS) |
|  |  | C50.01x | Malignant neoplasm of nipple and areola, female |
|  |  | C50.11x | Malignant neoplasm of central portion of breast |
|  |  | C50.21x | Malignant neoplasm of upper-inner quadrant of breast |
|  |  | C50.31x | Malignant neoplasm of lower-inner quadrant of breast |
|  |  | C50.41x | Malignant neoplasm of upper-outer quadrant of breast |
|  |  | C50.51x | Malignant neoplasm of lower-outer quadrant of breast |
|  |  | C50.61x | Malignant neoplasm of axillary tail of breast |
|  |  | C50.81x | Malignant neoplasm of overlapping sites of breast |
|  |  | C50.91x | Malignant neoplasm of breast of unspecified site |

**Abbreviations**: CPT, Current Procedural Terminology; ICD-10-CM, International Classification of Diseases, Tenth Revision, Clinical Modification.

**eTable 2. Construction of Study Cohorts**

| **Commercial** | | **Included** | **Excluded** | |
| --- | --- | --- | --- | --- |
|  | Females aged 18-64 who underwent BCS between 1/01/2017-12/31/2019 for breast cancer | 28,962 |  |  |
|  | Continuous enrollment 1 year before the index BCS | 23,125 | 5,837 |  |
|  | Continuous enrollment 1 year after the index BCS | 17,441 | 5,684 |  |
|  | No prior breast operation (lumpectomy or mastectomy) | 17,152 | 289 |  |
|  | No missing information | 17,129 | 23 |  |
|  | Final Study Participants | 17,129 |  |  |
| **Medicare** | |  |  |  |
|  | Females aged 18 and older who underwent BCS between 1/01/2017-10/31/2019 for breast cancer | 8,380 |  |  |
|  | Continuous enrollment 1 year before the index BCS | 7,452 | 928 |  |
|  | Continuous enrollment 1 year after the index BCS | 7,087 | 365 |  |
|  | No prior breast operation (lumpectomy or mastectomy) | 6,998 | 89 |  |
|  | No missing information | 6,977 | 21 |  |
|  | Final Study Participants | 6,977 |  |  |

**eTable 3. Codes to identify complications**

| **Category/ ICD-10-CM** | **Description** |
| --- | --- |
| **Infection** |  |
| N61.0 | Mastitis without abscess |
| N61.1 | Abscess of the breast and nipple |
| L02.21 | Cutaneous abscess of trunk |
| L02.22 | Furuncle of trunk |
| L02.23 | Carbuncle of trunk |
| T81.4 | Infection following a procedure |
| L03.31 | Cellulitis of trunk |
| L03.113 | Cellulitis of right upper limb |
| L03.114 | Cellulitis of left upper limb |
| T85.79 | Infection and inflammatory reaction due to other internal prosthetic devices, implants and grafts |
| T86.822 | Skin graft (allograft) (autograft) infection |
| **Breast reconstruction deformity and wound dehiscence** | |
| T85.4 | Mechanical complication of breast prosthesis and implant |
| N65 | Deformity and disproportion of reconstructed breast |
| S21.00 | Unspecified open wound of breast |
| T81.30 | Disruption of wound, unspecified |
| T81.31 | Disruption of external operation (surgical) wound, not elsewhere classified |
| T81.32 | Disruption of internal operation (surgical) wound, not elsewhere classified |
| T81.83 | Persistent postprocedural fistula |
| **Hemorrhage, hematoma, seroma** | |
| I97.42 | Intraoperative hemorrhage and hematoma of a circulatory system organ or structure complicating other procedure |
| I97.62 | Postprocedural hemorrhage, hematoma and seroma of a circulatory system organ or structure following other procedure |
| L76.0 | Intraoperative hemorrhage and hematoma of skin and subcutaneous tissue complicating a procedure |
| L76.2 | Postprocedural hemorrhage of skin and subcutaneous tissue following a procedure |
| L76.3 | Postprocedural hematoma and seroma of skin and subcutaneous tissue following a procedure |
| R58 | Hemorrhage, not elsewhere classified |
| **Breast pain** |  |
| N64.4 | Mastodynia |
| **Fat necrosis** |  |
| N64.1 | Fat necrosis of breast |
| **Other** |  |
| I97.1x1 | Other postprocedural cardiac functional disturbances following other surgery |
| I97.2 | Postmastectomy lymphedema syndrome |
| I97.3 | Postprocedural hypertension |
| I97.52 | Accidental puncture and laceration of a circulatory system organ or structure during other procedure |
| I97.711 | Intraoperative cardiac arrest during other surgery |
| I97.791 | Other intraoperative cardiac functional disturbances during other surgery |
| I97.811 | Intraoperative cerebrovascular infarction during other surgery |
| I97.821 | Postprocedural cerebrovascular infarction during other surgery |
| I97.88 | Other intraoperative complications of the circulatory system, not elsewhere classified |
| I97.89 | Other postprocedural complications and disorders of the circulatory system, not elsewhere classified |
| T81.89 | Other complications of procedures, not elsewhere classified |
| T81.9 | Unspecified complication of procedure |
| T88.8 | Other specified complications of surgical and medical care, not elsewhere classified |
| T88.9 | Complication of surgical and medical care, unspecified |
| L76.8 | Other intraoperative and postprocedural complications of skin and subcutaneous tissue |

**eTable 4. Codes to identify Charlson Comorbidities excluding breast cancer**

| **Conditions** | **ICD-10-CM** |
| --- | --- |
| Myocardial infarction | I21x, I22x, I25.2 |
| Congestive heart failure | I09.81, I11.0, I13.0, I13.2, I25.5, I42.0, I42.5 - I42.9, I43.x, I50.x |
| Peripheral vascular disease | A52.01, I70.x, I71.x, I73.1, I73.8x, I73.9, I77.1, I79.x, K55.1, K55.8, K55.9, Z95.82x |
| Cerebrovascular disease | H34.0x, G45.x, G46.x, I60.x - I69.x |
| Dementia | F01.x, F02.x, F03.x, G30.x, G31.0x, G31.1, G31.83, G31.84, R41.81 |
| Chronic pulmonary disease | I27.8x, I27.9, J40.x - J47.x, J60.x - J67.x, J68.4, J70.1, J70.3 |
| Rheumatic disease | M31.5, M32.x, M33.x, M34.x, M05.x, M06.x, M35.1, M35.3, M36.0 |
| Peptic ulcer disease | K25.x–K28.x |
| Mild liver disease | B18.x, K70.0, K70.1x, K70.2, K70.3x, K70.9, K71.3, K71.4, K71.5x, K71.7, K73.x, K74.x, K76.0, K76.2 - K76.4, K76.8x, K76.9, Z94.4 |
| Moderate or severe liver disease | I85.0x, I86.4, K70.4x, K71.1x, K72.1x, K72.9x, K76.5, K76.6, K76.7 |
| Diabetes without chronic complication | E10.1x, E10.6x, E10.8, E10.9, E11.0x, E11.1x, E11.6x, E11.8, E11.9, E13.0x, E13.1x, E13.6x, E13.8, E13.9 |
| Diabetes with chronic complication | E10.2x, E10.3x, E10.4x, E10.5x, E11.2x, E11.3x, E11.4x, E11.5x, E13.2x, E13.3x, E13.4x, E13.5x |
| Hemiplegia or paraplegia | G04.1, G11.4, G80.1, G80.2, G81.x, G82.x, G83.0x - G83.4x, G83.9 |
| Renal disease | I12.x, I13.x, N03.x, N05.2-N05.7, N18.x, N19.x, N25.0, Z94.0, Z99.2, Z49.x |
| Any malignancy, including lymphoma and leukemia, except malignant nonmelanoma neoplasm of skin | C0x.x, -C3x.x, C40.x, C41.x, C43.x C45.x-C49.x, C6x.x, C70.x-C76.x, C7A.x, C81.x-C86.x, C88.x, C9x.x |
| Metastatic solid tumor | C77.x-C80.x (except C79.8x) |
| AIDS/HIV | B20.x |

**eTable 5. CPT/HCPCS and revenue codes to identify the category of services**

| **Type** | **Codes** | **Descriptions** |
| --- | --- | --- |
| **Breast Surgery** | |  |
| CPT | 19000-19499 | Surgical procedures on the breast |
| CPT | L8000-L8039 | Breast Prosthetics and Accessories |
| CPT | 38525, 38530, 38740, 38745, 38792, 38900 | Surgical procedures on the lymph nodes and lymphatic channels in the breast area |
| CPT | 00100-01999 | Anesthesia^a^ |
| Revenue | 037X, 0963 | Anesthesia^a^ |
| Revenue | 036X, 0975 | Operating room^a^ |
| Revenue | 049X | Ambulatory surgical care^a^ |
| Revenue | 071X | Recovery room^a^ |
| CPT | 70010-77092, 78012-78999 | Diagnostic imaging^a^ |
| HCPCS | G0202, G0204, G0206 | Screening & diagnostic mammography^a^ |
| HCPCS | G0279 | Diagnostic digital breast tomosynthesis^a^ |
| Revenue | 032X, 0340, 0341, 0343, 035X, 040X, 061X, 0972 | Diagnostic imaging^a^ |
| DRG | 579, 580, 581 | Other, subcutaneous tissue and breast procedure^b^ |
| DRG | 582, 583, 584,585 | Breast biopsy and mastectomy |
| **Radiation, Therapeutic** | |  |
| CPT | 77261-77799 | Radiation oncology treatment |
| CPT | 79005-79999 | therapeutic nuclear medicine |
| HCPCS | G6001-G6017 | Radiation therapy services |
| Revenue | 0330, 0333, 0342, 0344, 0973 | Radiology therapeutic |
| **Chemotherapy** | |  |
| HCPCS | J9000-J9999, J8501-J8999 | Chemotherapy drugs |
| CPT | 96401-96549 | Chemotherapy administration |
| Revenue | 0331, 0332, 0335 | Chemotherapy administration |
| **Pathology** |  |  |
| CPT | 88000-89240 | Pathology procedures |
| Revenue | 031X | Laboratory pathology |
| **Laboratory** |  |  |
| CPT | 80047-87999, 89250-89398, 36415, 36416 | Laboratory procedures |
| Revenue | 030X, 0971 | Laboratory |
| **Radiation, Diagnostic** | |  |
| CPT | 70010-77092, 78012-78999 | Diagnostic imaging |
| HCPCS | G0202, G0204, G0206, G0279 | Screening & diagnostic imaging on breast |
| Revenue | 032X, 0340, 0341, 0343, 035X, 040X, 061X, 0972 | Diagnostic imaging |

**Abbreviations**: CPT, Current Procedural Terminology; HCPCS, Healthcare Common Procedure Coding System

^a^Restricted to the service that was billed together with CPT codes for breast surgery or occurred on the same date as the breast surgery.

^b^Restricted to the inpatient hospitalization with primary or secondary diagnoses for breast cancer (ICD-10-CM D05.x or C50.x).

**eTable 6. CPT/HCPCS Codes to identify oncoplasty and breast reconstruction**

| **Code** | **Description** |
| --- | --- |
| 11920-11922 | Tattooing, intradermal introduction of insoluble opaque pigments to correct color defects of skin |
| 11970 | Replacement of tissue expander with permanent implant |
| 11971 | Removal of tissue expander and replacement without permanent implant |
| 14000 | Adjacent tissue transfer or rearrangement, trunk; defect 10 sq cm or less |
| 14001 | Adjacent tissue transfer or rearrangement, trunk; defect 10.1 sq cm to 30.0 sq cm |
| 14301 | Adjacent tissue transfer or rearrangement, any area |
| 14302 | Adjacent tissue transfer or rearrangement, any area |
| 15771, 15772 | Grafting of autologous fat harvested by liposuction technique to trunk, breast, scalp, arms and or legs |
| 15777 | Implantation of biologic implant for soft tissue reinforcement (breast, trunk) |
| 15877 | Suction assisted lipectomy; trunk |
| 19316 | Mastopexy |
| 19318 | Breast Reduction |
| 19324 | Mammaplasty, augmentation; without prosthetic implant |
| 19325 | Breast augmentation with implant |
| 19328 | Removal of intact breast implant |
| 19330 | Removal of ruptured breast implant |
| 19340 | Insertion of breast implant on same day of mastectomy |
| 19342 | Insertion or replacement of breast implant on separate day from mastectomy |
| 19350 | Nipple/areola reconstruction |
| 19355 | Correction of inverted nipples |
| 19357 | Breast reconstruction, immediate or delayed, with tissue expander |
| 19361 | Breast reconstruction; with latissimus dorsi flap |
| 19364 | Breast reconstruction; with free flap (e.g., TRAM, DIEP, SIEA, GAP flap) |
| 19366 | Breast reconstruction with other technique |
| 19367, 19368 | Breast reconstruction; w/ single-pedicled TRAM flap |
| 19369 | Breast reconstruction; with bipedicled TRAM flap |
| 19370 | Revision of peri-implant capsule, breast |
| 19371 | Peri-implant capsulectomy, breast, complete, including removal of all intracapsular contents |
| 19380 | Revision of reconstructed breast |
| 19396 | Preparation of moulage for custom breast implant |
| C1789 | Prosthesis, breast (implantable) |
| L8600 | Implantable breast prosthesis, silicone or equal |
| S2066 | Breast reconstruction with GAP flap |
| S2067 | Breast reconstruction of a single breast with “stacked” DIEP flap(s) and/or GAP flap(s) |
| S2068 | Breast reconstruction with DIEP flap or SIEA flap |

**Abbreviations**: CPT, Current Procedural Terminology; HCPCS, Healthcare Common Procedure Coding System

**eTable 7. Reoperation rates by subgroup and risk ratios: Commercial cohort**

|  | **Reoperation, %** | **Crude RR** | **p-value** | **Adjusted RR^a^** | **p-value** |
| --- | --- | --- | --- | --- | --- |
| **All** | 21.1 (20.5-21.8) |  |  |  |  |
| **Age** |  |  |  |  |  |
| 18-44 | 26.5 (24.6-28.5) | 1.00 |  | 1.00 |  |
| 45-54 | 22.0 (21.0-23.0) | 0.83 (0.76-0.90) | <0.001 | 0.81 (0.74-0.88) | <0.001 |
| 55-64 | 19.4 (18.5-20.2) | 0.73 (0.67-0.79) | <0.001 | 0.71 (0.65-0.77) | <0.001 |
| **Diagnosis** |  |  |  |  |  |
| IBC | 18.0 (17.3-18.7) | 1.00 |  | 1.00 |  |
| DCIS | 30.8 (29.2-32.3) | 1.71 (1.61-1.82) | <0.001 | 1.62 (1.52-1.73) | <0.001 |
| Mixed | 22.9 (21.0-24.8) | 1.27 (1.16-1.39) | <0.001 | 1.24 (1.13-1.36) | <0.001 |
| **CCI** |  |  |  |  |  |
| 0-1 | 21.9 (21.2-22.6) | 1.00 |  | 1.00 |  |
| 2-5 | 19.2 (17.5-21.0) | 0.88 (0.80-0.97) | 0.009 | 0.93 (0.85-1.03) | 0.16 |
| >5 | 17.3 (15.4-19.1) | 0.79 (0.71-0.88) | <0.001 | 1.03 (0.91-1.16) | 0.64 |
| **NAC** |  |  |  |  |  |
| No | 22.2 (21.5-22.8) | 1.00 |  | 1.00 |  |
| Yes | 14.6 (13.1-16.0) | 0.66 (0.59-0.73) | <0.001 | 0.71 (0.64-0.80) | <0.001 |
| **Immediate OPS^b^** | |  |  |  |  |
| No | 22.0 (21.3-22.7) | 1.00 |  | 1.00 |  |
| Yes | 17.8 (16.5-19.0) | 0.81 (0.75-0.87) | <0.001 | 0.83 (0.77-0.89) | <0.001 |
| **Year** |  |  |  |  |  |
| 2017 | 21.4 (20.4-22.5) | 1.00 |  | 1.00 |  |
| 2018 | 21.8 (20.7-22.9) | 1.02 (0.95-1.09) | 0.63 | 1.01 (0.94-1.08) | 0.87 |
| 2019 | 20.2 (19.2-21.2) | 0.94 (0.88-1.01) | 0.097 | 0.94 (0.88-1.01) | 0.082 |
| **Region** |  |  |  |  |  |
| Northeast | 21.1 (19.8-22.4) | 1.00 |  | 1.00 |  |
| Midwest | 20.1 (18.8-21.4) | 0.95 (0.87-1.04) | 0.31 | 0.98 (0.89-1.07) | 0.63 |
| South | 21.6 (20.7-22.5) | 1.02 (0.95-1.10) | 0.53 | 1.06 (0.98-1.14) | 0.15 |
| West | 21.3 (19.6-22.9) | 1.01 (0.91-1.11) | 0.87 | 1.05 (0.95-1.16) | 0.34 |

**Abbreviations**: RR, risk ratio; IBC, invasive breast cancer; DCIS, ductal carcinoma in situ; CCI, Charlson Comorbidity Index; NAC, neoadjuvant chemotherapy; OPS, oncoplastic surgery.

^a^Adjusted risk ratios were reported from multivariable modified Poisson regression models including age, cancer diagnosis, CCI, NAC, immediate OPS, index year and census region.

^a^Immediate OPS was defined as OPS on the same date of index BCS as the breast surgery.

**eTable 8. Reoperation rates by subgroup and risk ratios: Medicare cohort**

|  | **Reoperation, %** | **Crude RR** | **p-value** | **Adjusted RR^a^** | **p-value** |
| --- | --- | --- | --- | --- | --- |
| **All** | 14.9 (14.1-15.7) |  |  |  |  |
| **Age** |  |  |  |  |  |
| 18-64 | 18.6 (14.4-22.7) | 1.00 |  | 1.00 |  |
| 65-74 | 15.5 (14.4-16.7) | 0.84 (0.66-1.06) | 0.13 | 0.86 (0.68-1.10) | 0.23 |
| 75-84 | 14.3 (12.9-15.7) | 0.77 (0.60-0.98) | 0.036 | 0.81 (0.63-1.03) | 0.09 |
| 85+ | 10.4 (07.8-13.0) | 0.56 (0.40-0.78) | 0.001 | 0.61 (0.43-0.86) | 0.005 |
| **Diagnosis** |  |  |  |  |  |
| IBC | 12.7 (11.8-13.6) | 1.00 |  | 1.00 |  |
| DCIS | 24.0 (21.4-26.6) | 1.89 (1.66-2.14) | <0.001 | 1.80 (1.58-2.05) | <0.001 |
| Mixed | 18.5 (15.2-21.8) | 1.45 (1.20-1.76) | <0.001 | 1.42 (1.17-1.72) | <0.001 |
| **CCI** |  |  |  |  | 0.35 |
| 0-1 | 15.6 (14.4-16.7) | 1.00 |  | 1.00 |  |
| 2-5 | 14.2 (12.8-15.6) | 0.91 (0.81-1.03) | 0.15 | 0.95 (0.84-1.08) | 0.44 |
| >5 | 13.6 (11.1-16.0) | 0.87 (0.72-1.06) | 0.17 | 1.03 (0.84-1.25) | 0.80 |
| **NAC** |  |  |  |  |  |
| No | 15.3 (14.4-16.2) | 1.00 |  | 1.00 |  |
| Yes | 09.4 (06.8-12.0) | 0.62 (0.46-0.82) | 0.001 | 0.65 (0.48-0.88) | 0.005 |
| **Immediate OPS^b^** | |  |  |  |  |
| No | 15.1 (14.1-16.0) | 1.00 |  | 1.00 |  |
| Yes | 14.1 (12.1-16.0) | 0.93 (0.80-1.09) | 0.38 | 0.95 (0.81-1.11) | 0.50 |
| **Year** |  |  |  |  |  |
| 2017 | 15.7 (14.2-17.1) | 1.00 |  | 1.00 |  |
| 2018 | 14.7 (13.3-16.1) | 0.94 (0.82-1.07) | 0.35 | 0.94 (0.82-1.08) | 0.38 |
| 2019 | 14.3 (12.9-15.8) | 0.91 (0.80-1.05) | 0.21 | 0.93 (0.81-1.06) | 0.27 |
| **Region** |  |  |  |  |  |
| Northeast | 13.1 (11.3-14.9) | 1.00 |  | 1.00 |  |
| Midwest | 16.8 (15.0-18.7) | 1.28 (1.08-1.53) | 0.005 | 1.27 (1.07-1.51) | 0.007 |
| South | 14.8 (13.5-16.2) | 1.13 (0.96-1.33) | 0.14 | 1.13 (0.96-1.33) | 0.13 |
| West | 14.7 (12.8-16.5) | 1.12 (0.93-1.35) | 0.24 | 1.15 (0.96-1.39) | 0.13 |
| **Race/ethnicity** | |  |  |  |  |
| White | 14.5 (13.6-15.4) | 1.00 |  | 1.00 |  |
| Black | 19.1 (15.7-22.4) | 1.31 (1.09-1.58) | 0.004 | 1.20 (0.99-1.45) | 0.06 |
| Hispanic | 11.4 (07.2-15.6) | 0.79 (0.54-1.14) | 0.21 | 0.77 (0.53-1.12) | 0.18 |
| Other | 17.3 (12.8-21.9) | 1.19 (0.91-1.56) | 0.20 | 1.13 (0.87-1.48) | 0.35 |

**Abbreviations**: RR, risk ratio; IBC, invasive breast cancer; DCIS, ductal carcinoma in situ; CCI, Charlson Comorbidity Index; NAC, neoadjuvant chemotherapy; OPS, oncoplastic surgery.

^a^Adjusted risk ratios were reported from multivariable modified Poisson regression models including age, cancer diagnosis, CCI, NAC, immediate OPS, index year and census region.

^a^Immediate OPS was defined as OPS on the same date of index BCS as the breast surgery.

**eTable 9. Complication rates** **by subgroup and risk ratios: Commercial cohort**

|  | **Complications, %** | **Crude RR** | **p-value** | **Adjusted RR^a^** | **p-value** |
| --- | --- | --- | --- | --- | --- |
| **All** | 20.2 (19.6-20.8) |  |  |  |  |
| **Reoperation** | |  |  |  |  |
| No | 17.7 (17.1-18.3) | 1.00 |  | 1.00 |  |
| Repeated BCS | 28.7 (27.0-30.5) | 1.62 (1.51-1.74) | <0.001 | 1.66 (1.55-1.78) | <0.001 |
| Conversion to Mastectomy | 31.8 (29.0-34.6) | 1.80 (1.63-1.98) | <0.001 | 1.30 (1.18-1.44) | <0.001 |
| **Age** |  |  |  |  |  |
| 18-44 | 23.1 (21.3-25.0) | 1.00 |  | 1.00 |  |
| 45-54 | 21.1 (20.0-22.1) | 0.91 (0.83-1.00) | 0.051 | 0.98 (0.89-1.07) | 0.67 |
| 55-64 | 19.0 (18.2-19.8) | 0.82 (0.75-0.90) | <0.001 | 0.93 (0.85-1.02) | 0.13 |
| **Diagnosis** |  |  |  |  |  |
| IBC | 19.9 (19.2-20.7) | 1.00 |  | 1.00 |  |
| DCIS | 20.5 (19.2-21.8) | 1.03 (0.95-1.11) | 0.48 | 1.02 (0.95-1.10) | 0.532 |
| Mixed | 21.4 (19.5-23.2) | 1.07 (0.98-1.18) | 0.14 | 1.04 (0.95-1.14) | 0.351 |
| **CCI** |  |  |  |  |  |
| 0-1 | 19.2 (18.5-19.8) | 1.00 |  | 1.00 |  |
| 2-5 | 22.2 (20.4-24.1) | 1.16 (1.06-1.27) | 0.001 | 1.19 (1.09-1.30) | <0.001 |
| >5 | 26.8 (24.6-29.0) | 1.40 (1.28-1.53) | <0.001 | 1.28 (1.16-1.42) | <0.001 |
| **NAC** |  |  |  |  |  |
| No | 19.4 (18.8-20.0) | 1.00 |  | 1.00 |  |
| Yes | 25.5 (23.7-27.2) | 1.31 (1.21-1.42) | <0.001 | 1.16 (1.07-1.27) | 0.001 |
| **OPS** | |  |  |  |  |
| No | 15.5 (14.9-16.1) | 1.00 |  | 1.00 |  |
| Yes | 32.8 (31.5-34.2) | 2.12 (2.00-2.24) | <0.001 | 2.04 (1.92-2.16) | <0.001 |
| **Year** |  |  |  |  |  |
| 2017 | 18.1 (17.0-19.1) | 1.00 |  | 1.00 |  |
| 2018 | 21.2 (20.1-22.3) | 1.18 (1.09-1.27) | <0.001 | 1.14 (1.06-1.23) | <0.001 |
| 2019 | 21.3 (20.3-22.4) | 1.18 (1.10-1.27) | <0.001 | 1.17 (1.09-1.26) | <0.001 |
| **Region** |  |  |  |  |  |
| Northeast | 18.0 (16.8-19.3) | 1.00 |  | 1.00 |  |
| Midwest | 18.7 (17.4-20.0) | 1.04 (0.94-1.14) | 0.46 | 1.06 (0.96-1.16) | 0.24 |
| South | 22.0 (21.0-22.9) | 1.22 (1.12-1.32) | <0.001 | 1.19 (1.10-1.29) | <0.001 |
| West | 20.4 (18.8-22.1) | 1.13 (1.02-1.26) | 0.02 | 1.06 (0.96-1.18) | 0.25 |

**eTable 10. Complication rates** **by subgroup and risk ratios: Medicare cohort**

|  | **Complications, %** | **Crude RR** | **p-value** | **Adjusted RR^a^** | **p-value** |
| --- | --- | --- | --- | --- | --- |
| **All** | 18.8 (17.9-19.8) |  |  |  |  |
| **Reoperation** | |  |  |  |  |
| No | 16.4 (15.4-17.3) | 1.00 |  | 1.00 |  |
| Repeated BCS | 30.0 (26.7-33.2) | 1.83 (1.62-2.07) | <0.001 | 1.82 (1.61-2.06) | <0.001 |
| Conversion to Mastectomy | 41.0 (35.2-46.9) | 2.51 (2.15-2.93) | <0.001 | 2.07 (1.77-2.44) | <0.001 |
| **Age** |  |  |  |  |  |
| 18-64 | 26.5 (21.8-31.2) | 1.00 |  | 1.00 |  |
| 65-74 | 19.7 (18.4-21.0) | 0.74 (0.61-0.90) | <0.001 | 0.81 (0.67-0.98) | 0.03 |
| 75-84 | 17.4 (15.9-19.0) | 0.66 (0.54-0.80) | <0.001 | 0.73 (0.60-0.89) | 0.002 |
| 85+ | 13.5 (10.5-16.4) | 0.51 (0.38-0.67) | <0.001 | 0.61 (0.46-0.81) | 0.001 |
| **Race/ethnicity** | |  |  |  |  |
| White | 18.5 (17.6-19.5) | 1.00 |  | 1.00 |  |
| Black | 21.3 (17.8-24.8) | 1.15 (0.97-1.36) | 0.11 | 1.02 (0.86-1.22) | 0.82 |
| Hispanic | 22.8 (17.3-28.4) | 1.23 (0.96-1.58) | 0.10 | 1.17 (0.91-1.49) | 0.22 |
| Other | 16.6 (12.2-21.0) | 0.90 (0.68-1.18) | 0.43 | 0.85 (0.65-1.12) | 0.26 |
| **Diagnosis** |  |  |  |  |  |
| IBC | 18.5 (17.5-19.6) | 1.00 |  | 1.00 |  |
| DCIS | 18.7 (16.3-21.0) | 1.01 (0.88-1.15) | 0.92 | 0.93 (0.81-1.07) | 0.327 |
| Mixed | 21.9 (18.4-25.5) | 1.18 (1.00-1.40) | 0.053 | 1.16 (0.98-1.37) | 0.094 |
| **CCI** |  |  |  |  |  |
| 0-1 | 17.8 (16.6-19.0) | 1.00 |  | 1.00 |  |
| 2-5 | 19.2 (17.6-20.8) | 1.08 (0.97-1.20) | 0.16 | 1.11 (0.99-1.23) | 0.06 |
| >5 | 22.8 (19.8-25.8) | 1.28 (1.11-1.48) | <0.001 | 1.25 (1.07-1.45) | <0.001 |
| **NAC** |  |  |  |  |  |
| No | 18.6 (17.7-19.6) | 1.00 |  | 1.00 |  |
| Yes | 21.6 (17.9-25.3) | 1.16 (0.97-1.39) | 0.11 | 1.07 (0.89-1.28) | 0.48 |
| **OPS** |  |  |  |  |  |
| No | 16.1 (15.2-17.1) | 1.00 |  | 1.00 |  |
| Yes | 30.6 (28.1-33.1) | 1.90 (1.72-2.10) | <0.001 | 1.75 (1.58-1.94) | <0.001 |
| **Year** |  |  |  |  |  |
| 2017 | 18.7 (17.1-20.3) | 1.00 |  | 1.00 |  |
| 2018 | 19.7 (18.2-21.3) | 1.05 (0.94-1.18) | 0.38 | 1.06 (0.95-1.19) | 0.28 |
| 2019 | 17.9 (16.3-19.5) | 0.95 (0.84-1.08) | 0.46 | 0.96 (0.85-1.08) | 0.50 |
| **Region** |  |  |  |  |  |
| Northeast | 17.0 (15.0-19.0) | 1.00 |  | 1.00 |  |
| Midwest | 19.1 (17.1-21.0) | 1.12 (0.96-1.31) | 0.15 | 1.12 (0.96-1.30) | 0.15 |
| South | 19.5 (18.0-21.0) | 1.15 (1.00-1.32) | 0.06 | 1.11 (0.97-1.28) | 0.12 |
| West | 19.0 (17.0-21.1) | 1.12 (0.95-1.31) | 0.17 | 1.06 (0.91-1.24) | 0.43 |


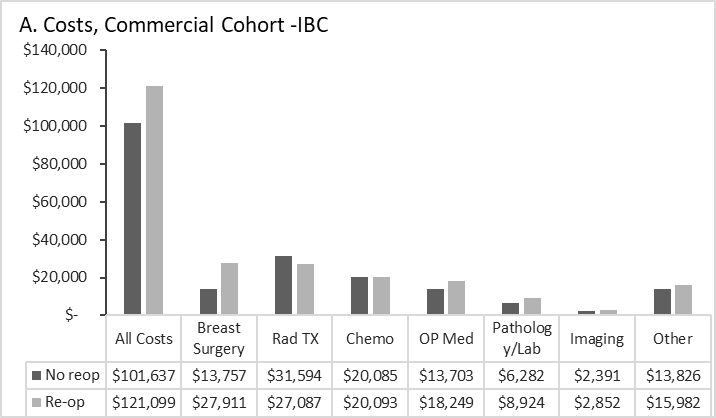

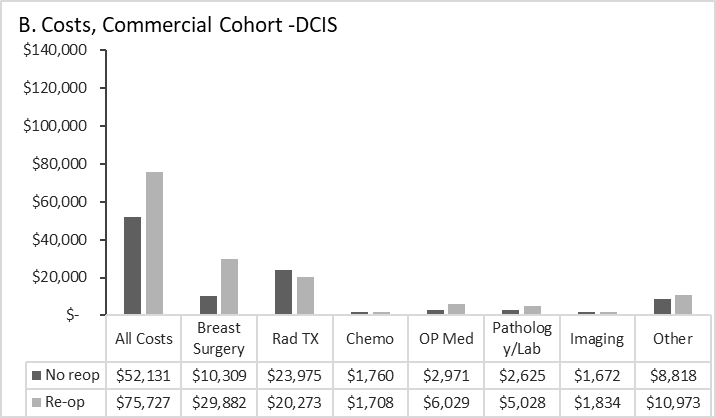

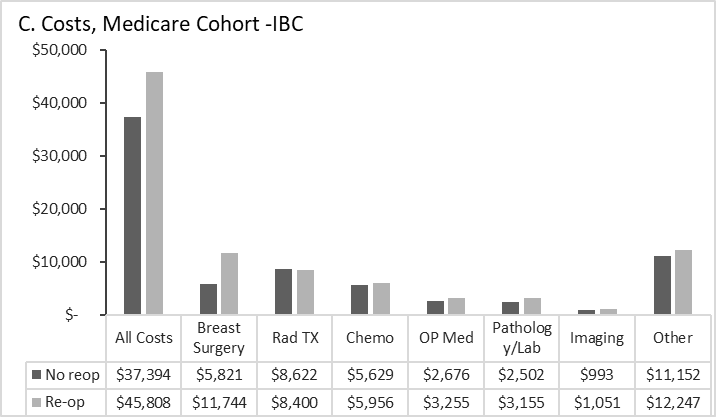

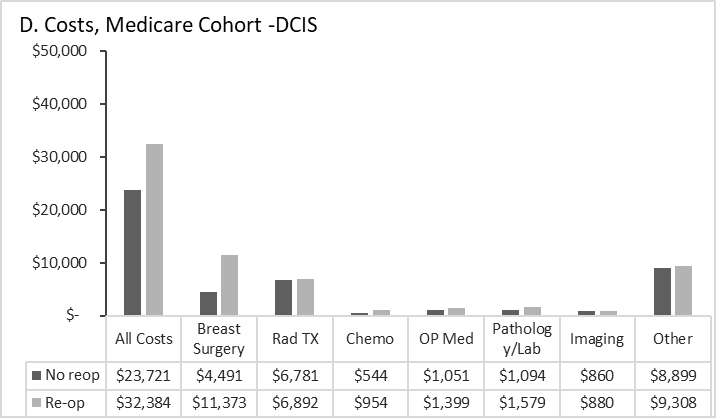


**eFigure 1. Costs by Reoperation Status and Service type**. Mean annual healthcare costs were aggregated by service type stratified by reoperation status. Breast surgery included inpatient, outpatient and professional claims related to surgical procedures on the breast. Radiation treatment included radiation oncology treatment and other radiology treatment. OP Med indicates medication administered at the outpatient setting and included ancillary treatment during chemotherapy treatment and hormonal/immunology medication as well. IBC indicates invasive breast cancer; DCIS, ductal carcinoma in situ.
